# Machine Learning-Based Process Optimization in Biopolymer Manufacturing: A Review

**DOI:** 10.3390/polym16233368

**Published:** 2024-11-29

**Authors:** Ivan Malashin, Dmitriy Martysyuk, Vadim Tynchenko, Andrei Gantimurov, Andrey Semikolenov, Vladimir Nelyub, Aleksei Borodulin

**Affiliations:** 1 Bauman Moscow State Technical University, 105005 Moscow, Russia; dmart9945@mail.ru (D.M.);; 2 Far Eastern Federal University, 690922 Vladivostok, Russia

**Keywords:** ML, biopolymers, process optimization, materials science

## Abstract

The integration of machine learning (ML) into material manufacturing has driven advancements in optimizing biopolymer production processes. ML techniques, applied across various stages of biopolymer production, enable the analysis of complex data generated throughout production, identifying patterns and insights not easily observed through traditional methods. As sustainable alternatives to petrochemical-based plastics, biopolymers present unique challenges due to their reliance on variable bio-based feedstocks and complex processing conditions. This review systematically summarizes the current applications of ML techniques in biopolymer production, aiming to provide a comprehensive reference for future research while highlighting the potential of ML to enhance efficiency, reduce costs, and improve product quality. This review also shows the role of ML algorithms, including supervised, unsupervised, and deep learning algorithms, in optimizing biopolymer manufacturing processes.

## 1. Introduction

Biopolymers have garnered significant attention as sustainable alternatives to petroleum-based plastics due to their biodegradability and reduced environmental impact [1]. With the increasing awareness of environmental issues, industries are actively seeking ways to reduce plastic waste [2,3], and biopolymers have emerged as a key solution [4]. They are derived from renewable biological sources such as plants [5], algae [6], and microorganisms [7], offering a promising path to reduce the reliance on fossil fuels and mitigate the growing problem of plastic pollution. Their biodegradability is an advantage, as it ensures that these materials can break down into non-toxic components in the environment, contributing to a more sustainable and circular economy. As a result, biopolymers are finding expanding applications across various sectors, such as packaging [8], agriculture [9], automotive [10], textiles [11], and biomedical fields [12], where environmental sustainability and product performance are equally important.

The growing demand for biopolymers [13,14] has prompted the need for efficient, scalable manufacturing processes. However, the production of biopolymers presents several challenges that need to be addressed to fully capitalize on their potential. The manufacturing process involves complex biochemical reactions, including fermentation [15,16,17,18,19,20,21,22,23], polymerization [7,24,25,26,27,28,29,30,31,32], and extraction [33,34,35,36,37,38,39,40,41,42], which are highly sensitive to variations in raw material quality, environmental conditions, and process parameters. Even minor fluctuations in temperature [43,44,45,46,47], pH levels [48,49,50,51,52], or nutrient concentrations [9,53,54,55,56] during fermentation can significantly affect the yield and properties of the final product. Similarly, inconsistencies in raw materials [57,58,59,60,61]—such as variations in the composition of feedstocks or impurities—can disrupt polymerization and extraction processes, leading to inefficiencies, increased waste, and deviations from desired product specifications.

These fluctuations not only pose challenges for maintaining consistent product quality but also hinder scalability, as small-scale optimization techniques often fail to account for the variability encountered in large-scale operations [62]. Furthermore, the growing emphasis on sustainability compounds these challenges. Manufacturers must ensure that fluctuations do not compromise the eco-friendliness of the process, requiring continuous monitoring, precise control systems, and innovations in workflow optimization to minimize resource consumption, reduce emissions, and manage waste effectively [63,64,65,66,67,68,69,70,71,72]. Balancing these technical and environmental demands is crucial to achieving both production efficiency and long-term sustainability goals.

**Figure 1:**
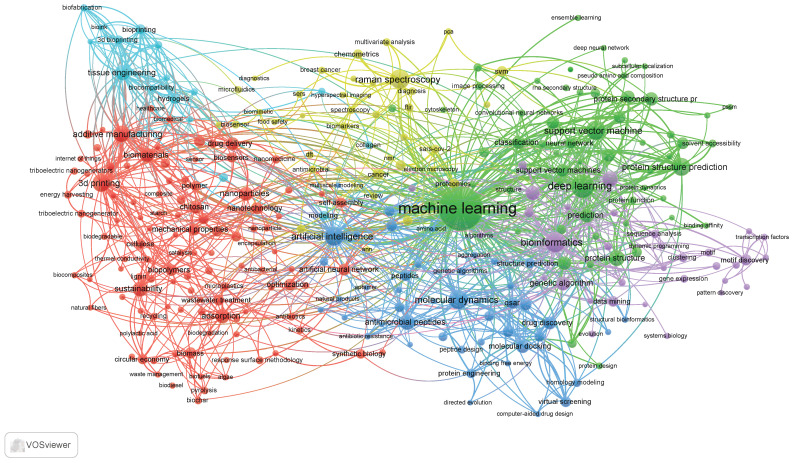
Keyword map from articles on the application of ML in biopolymers.

Machine learning (ML) offers promising solutions to these challenges by enabling real-time data processing [73], predictive modeling [74], and optimization of production workflows [75]. The integration of ML in biopolymer manufacturing can facilitate more precise control over the various stages of production, improving the consistency and quality of the final product. ML-driven predictive models can anticipate potential variations in raw material quality or environmental conditions [76,77], allowing manufacturers to adjust their processes proactively to maintain efficiency and sustainability. Moreover, ML can help to optimize energy consumption [78], reduce waste [79], and streamline logistics [80], further enhancing the overall sustainability of biopolymer production. ML algorithms, for instance, can analyze vast datasets from production lines to identify patterns and suggest process improvements, while ML-powered sensors [81] can monitor real-time performance metrics to ensure optimal operating conditions. A keyword map (Figure 1) was created using VOSviewer to analyze the scientific literature on the application of ML in biopolymers. This visualization highlights key topics, research directions, and their interconnections, providing insights into current trends and promising areas of study.

ML has already made a significant impact in fields such as materials science and chemical engineering, and its transformative potential in biopolymers is becoming increasingly evident. For example, in materials science, ML has been used to predict material properties [82,83,84,85,86,87,88,89,90], optimize the design of alloys [84,90,91,92,93,94,95,96,97,98], and accelerate the discovery of novel materials [99,100,101,102,103,104,105,106,107,108]. Building on tools like ab initio calculations, density functional theory (DFT), and quantum chemistry databases, ML integrates computational and experimental approaches, facilitating breakthroughs in catalyst design, energy materials, and drug discovery [109].

The application of ML to biopolymers faces several challenges. Data quality and availability are key issues, as biopolymer systems involve complex variables and often suffer from sparse, noisy, or incomplete datasets, leading to overfitting or unreliable predictions [72,110,111,112,113,114]. Additionally, the nonlinear and multifactorial nature of biopolymer processes (e.g., fermentation, polymerization, extraction) complicates modeling with existing ML algorithms, which may assume simpler linear relationships [115,116,117,118]. Interpretability [119,120,121,122,123,124,125,126,127,128] is another challenge, as many ML models, especially deep learning, are seen as “black boxes”, making it difficult to understand the reasons behind predictions. This lack of transparency hinders trust and limits adoption in industrial settings where clear explanations are essential. ML models also struggle with scalability and generalization [128,129,130,131,132,133,134,135,136,137]; those trained on small-scale data often fail to perform well in larger, more variable real-world environments, restricting their broader application. Finally, the computational requirements [138,139,140,141,142,143,144,145,146,147] of advanced ML models can be a barrier for researchers and small manufacturers lacking access to high-performance computing resources, further complicating real-time model deployment in biopolymer production.

Recent reviews on biopolymers and the application of ML underscore the growing need for innovative approaches to address challenges associated with variable feedstock properties and complex manufacturing processes. These studies highlight the potential of ML to optimize key production stages, enhance sustainability, and improve the quality of final products across various industries.

In tissue engineering, significant advancements over the past 30 years have positioned additive manufacturing (AM) as a tool for replacing and regenerating injured tissues. AM, particularly 3D printing, is recognized as an effective method for synthesizing conducting polymer-based materials in applications such as flexible electronics, bioelectronics, and energy storage [148]. Das et al. [149] focus on gelatin methacryloyl (GelMA) as a versatile biomaterial for 3D bioprinting, discussing strategies to optimize its rheological, mechanical, and biophysical properties. Their review also explores the potential integration of artificial intelligence (AI) and ML to predict printability and functionality for clinically relevant applications, emphasizing the transformative role of computational tools in advancing tissue engineering solutions.

Similarly, the rising global prevalence of diabetes calls for innovative solutions, with hydrogel-based systems emerging as promising alternatives for non-invasive diagnosis, management, and treatment. Rahmani et al. [150] review the potential of hydrogels, their integration with Internet of Things (IoT) and ML technologies, and their role in advancing personalized and proactive diabetes care. These technologies offer new opportunities to improve patient outcomes through continuous monitoring and tailored therapeutic approaches.

In the food industry, consumer demand for safe and high-quality meat has driven the development of anthocyanin-based materials for real-time freshness monitoring. Xiong et al. [151] review recent advances in anthocyanin-based films, hydrogels, aerogels, and colorimetric sensor arrays. Their work highlights the potential of these materials to provide intuitive color signals indicating spoilage, while also addressing challenges related to sensitivity, stability, and integration with technologies like smartphones and ML for enhanced performance.

Furthermore, the integration of ML with algae-derived biopolymers, such as alginate and carrageenan, is transforming 3D printing by enabling sustainable and efficient additive manufacturing processes. Bin et al. [152] discuss advancements and challenges in this field, emphasizing ML’s role in optimizing material selection, predictive modeling, and quality control. Their findings demonstrate how the combination of ML and algae-based biopolymers can enhance mechanical properties and expand applications, particularly in areas such as bone tissue engineering.

The existing reviews highlight ML’s potential in biopolymer applications but overlook its specific role in optimizing manufacturing processes. A focused review is needed to address how ML can tackle challenges like feedstock variability, process efficiency, and product quality in biopolymer production.

This review aims to provide an overview of ML’s applications in the biopolymer manufacturing industry, outlining the potential of various ML techniques in improving production efficiency and sustainability. It explores key ML methodologies such as supervised and unsupervised learning, and how they are being utilized to address specific challenges in biopolymer production. Additionally, the review considers the future outlook of ML in the biopolymer industry, examining the potential for further innovations that could drive cost reductions, enhance material properties, and facilitate the development of new, advanced biopolymers that meet the demands of an evolving global market. To provide a clear structure, this review is organized as follows. It begins with an overview of biopolymer manufacturing (Section 2), covering key stages such as feedstock selection (Section 2.1), fermentation (Section 2.2), polymerization and extraction (Section 2.3), and quality control with post-processing techniques (Section 2.4). The discussion then shifts to the role of ML techniques in process optimization (Section 3), detailing approaches such as supervised learning (Section 3.1), unsupervised learning (Section 3.2), and neural networks with deep learning applications (Section 3.3). Challenges associated with integrating ML into biopolymer research are outlined in Section 4, followed by an exploration of future directions for advancing ML-driven solutions in this field (Section 5).

## 2. Overview of Biopolymer Manufacturing

Biopolymers are polymers derived from natural sources, including plants [153,154], animals [155,156], and microorganisms [7,157]. Unlike conventional petroleum-based plastics, biopolymers are sourced from renewable materials [158,159,160], which makes them highly attractive in the push for sustainable materials. They are biodegradable or compostable in many cases, reducing environmental impact and waste accumulation. Common types of biopolymers include polyhydroxyalkanoates (PHAs) [161,162], polylactic acid (PLA) [163,164], and starch-based polymers, each with unique properties suitable for various industrial applications. For instance, PHAs are microbial biopolymers that can be customized for diverse applications, while PLA, derived from fermented plant sugars, is widely used in packaging due to its favorable mechanical properties and biodegradability. Starch-based polymers, produced from plant-based starch, are often employed in food packaging and agricultural films.

ML is increasingly applied in biopolymer manufacturing to optimize the design, synthesis, and processing of biopolymers across various industries. By analyzing complex datasets, ML techniques can predict and enhance the functional properties of biopolymers, such as biodegradability, mechanical strength, and stability, enabling more efficient production processes, material selection, and product customization for applications in food, pharmaceuticals, packaging, and biomedical fields.

The production process of biopolymers typically involves several key stages that require careful control and optimization to ensure the quality and sustainability of the final product.

### 2.1. Feedstock Selection

The use of traditional food-based feedstocks like corn starch [165] or sugarcane [166] has been widely adopted due to their high availability and consistent quality, making them suitable for the large-scale production of biopolymers such as polylactic acid (PLA). However, these feedstocks can compete with food crops for arable land and water resources, raising concerns about their impact on food security and land use [167]. This has led to an increased interest in using non-food biomass as an alternative, including agricultural residues (such as corn stover, wheat straw, or rice husks), forestry waste, and industrial by-products. These materials offer a more sustainable option, as they do not compete directly with food production and often represent waste streams that would otherwise go unused or be disposed of.

Using non-food biomass can reduce the overall environmental impact of biopolymer production [168]. For instance, agricultural residues that are left over after crop harvesting, such as straw or husks, can be collected and processed into valuable feedstocks for biopolymer production without requiring additional land or resources [169]. Similarly, industrial by-products, such as lignin from paper production or glycerol from biodiesel manufacturing, can be repurposed, reducing waste and improving the circularity of industrial processes [170]. This not only minimizes the carbon footprint associated with raw material extraction but also enhances the sustainability of biopolymer production by diverting waste from landfills or incineration.

However, the quality of these alternative feedstocks can vary significantly due to factors such as geographic location [171], climate [39], and farming practices [64]. For example, agricultural residues from different regions or seasons may have different moisture content, fiber composition, or levels of impurities, all of which can affect the efficiency of fermentation and polymerization processes [172]. Climate conditions such as drought or excessive rainfall can also alter the chemical composition of plants, leading to fluctuations in the availability of sugars or starches that are essential for biopolymer production [173]. In regions with inconsistent farming practices or where crop management varies, the feedstock may require additional pretreatment steps, such as cleaning or fractionation, to ensure it meets the quality standards necessary for biopolymer synthesis [174].

**Figure 2:**
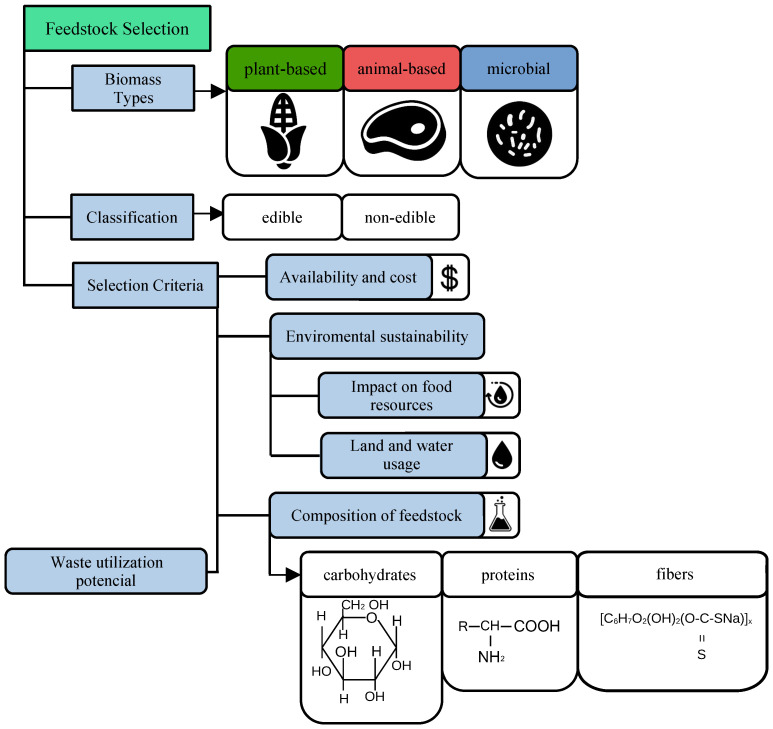
Biopolymer feedstock selection and sustainability considerations.

Furthermore, the supply chain logistics of sourcing feedstock from agricultural waste or non-food biomass can be complex [175]. Collecting, transporting, and storing large quantities of biomass often present logistical challenges due to the bulkiness and seasonal availability of these materials. In some cases, the energy required to process and transport low-density biomass can offset the environmental benefits of using renewable feedstocks [176]. To address these challenges, advancements in biorefining technologies are being developed to improve the conversion efficiency of various feedstocks into polymer precursors and optimize the overall sustainability of the production process [177]. Figure 2 is a visual representation of the key factors related to biopolymer feedstock selection and sustainability consideration.

### 2.2. Fermentation

In the fermentation stage, microorganisms such as bacteria [178], yeast [179], or fungi [180] convert renewable feedstocks into polymer precursors, which are the building blocks of biopolymers. This bioconversion process is highly dependent on the metabolic capabilities of the selected microorganisms and the specific fermentation conditions. The microorganisms used in fermentation act as biocatalysts, breaking down the feedstock—typically composed of sugars, starches, or lipids—into simpler molecules such as organic acids or alcohols. These precursor molecules are then used in subsequent steps to produce various biopolymers [181,182].

For example, in the production of polylactic acid (PLA), specific strains of lactic acid bacteria (e.g., *Lactobacillus* spp.) are employed to ferment sugars derived from feedstocks such as corn, sugarcane, or other carbohydrates [183]. During fermentation, these bacteria metabolize the sugars and convert them into lactic acid, a key precursor for PLA production [183]. Once lactic acid is produced, it undergoes polymerization to form PLA, a biodegradable thermoplastic that is widely used in packaging, disposable cutlery, and biomedical applications.

Similarly, polyhydroxyalkanoates (PHAs) are another class of biopolymers produced through microbial fermentation. In PHA production, bacteria such as Cupriavidus necator or Ralstonia eutropha are typically used to ferment sugars, lipids, or other organic substrates [184,185,186]. Under conditions of nutrient limitation (such as nitrogen or phosphorus deficiency), these bacteria accumulate PHAs intracellularly as carbon and energy storage compounds. The PHAs are then harvested from the bacterial cells and processed into biodegradable plastics with a range of applications, from packaging materials to medical devices.

The selection of the microorganism strain is perhaps the most important factor in fermentation. Different strains have varying metabolic pathways, growth rates, and tolerance to environmental conditions. For instance, some bacterial strains may be more efficient at converting specific feedstocks into lactic acid or PHAs, while others may be more resistant to by-products that could inhibit fermentation [187]. Genetic engineering and synthetic biological techniques have also enabled the development of engineered microbial strains with enhanced fermentation capabilities [188]. These engineered strains can exhibit higher productivity, increased substrate versatility, and reduced by-product formation, all of which contribute to a more efficient fermentation process.

The composition and quality of the feedstock used in fermentation affect the efficiency of microbial activity [189]. Feedstocks rich in fermentable sugars, such as glucose, fructose, or sucrose, are often preferred for high-yield lactic acid or PHA production. However, feedstocks can vary in their composition, particularly when derived from agricultural residues or industrial by-products [190]. Complex feedstocks may contain inhibitors such as lignin, phenolic compounds, or heavy metals, which can slow down microbial growth or reduce the yield of polymer precursors [191]. To address these challenges, pretreatment processes such as enzymatic hydrolysis, acid or alkali treatments, or steam explosion may be required to break down complex biomass into fermentable sugars, enhancing the efficiency of fermentation [192].

The fermentation environment impacts the determination of the productivity of microorganisms. Maintaining optimal conditions for temperature [193], pH [194], and oxygen levels (in the case of aerobic fermentation) is essential for maximizing microbial growth and metabolic activity [195]. Each microorganism strain has a specific range of temperature and pH where it performs optimally. For instance, the Lactobacillus species used in lactic acid production typically thrive at moderately acidic pH values (around pH 5–6) and temperatures ranging from 30 to 40 °C [196,197]. Deviations from these optimal conditions can slow down fermentation, reduce yields, or lead to the formation of unwanted by-products such as acetic acid or ethanol. In some cases, the process may need to be aerobic [198] (with oxygen) or anaerobic [199] (without oxygen), depending on the metabolic pathway of the microorganism. Therefore, precise control over these variables is crucial for achieving efficient biopolymer precursor production.

The duration of fermentation also affects the final yield of polymer precursors. Longer fermentation times may lead to higher yields but can increase operational costs and energy consumption [200]. Conversely, shorter fermentation times can reduce costs but may result in lower yields if microbial growth and metabolism are not fully optimized [201]. Batch, fed-batch, and continuous fermentation processes are commonly used to balance yield and efficiency. In a batch fermentation, the feedstock is added at the beginning, and the process runs until completion [202]. Fed-batch fermentation allows for the gradual addition of feedstock, enabling better control over microbial growth and product formation [203]. Continuous fermentation, on the other hand, enables the constant input of feedstock and removal of products, allowing for more consistent yields and higher productivity over time [204].

During fermentation, unwanted by-products can form due to suboptimal conditions or microbial metabolic pathways [205]. These by-products, such as acetic acid, ethanol, or hydrogen sulfide, can inhibit microbial growth, lower the yield of polymer precursors, and increase the need for costly purification steps. By optimizing the fermentation conditions—such as adjusting nutrient concentrations, pH levels, or oxygen availability—manufacturers can minimize by-product formation and improve the efficiency of precursor production. The metabolic engineering of microorganisms is also being used to reprogram metabolic pathways, reducing the production of by-products and improving the conversion efficiency of feedstock into biopolymer precursors [206].

**Figure 3:**
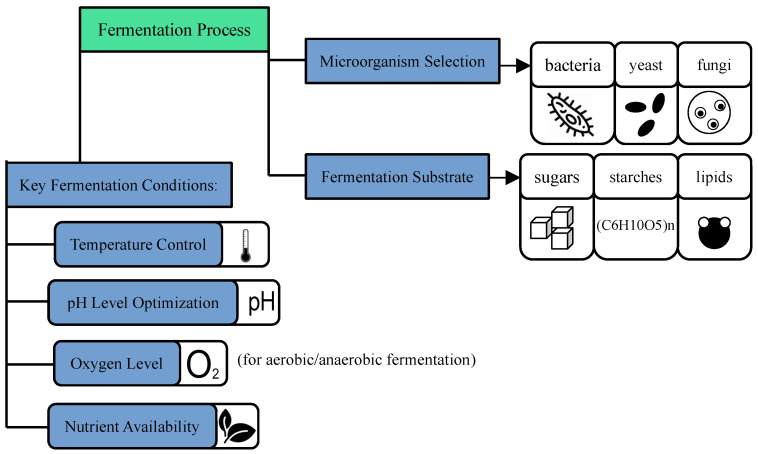
Key factors influencing fermentation in biopolymer production.

Given the complexity of microbial fermentation, maintaining optimal conditions is crucial for maximizing the yield of polymer precursors and minimizing the formation of by-products. Advances in bioprocess monitoring and control technologies are helping to improve the precision of fermentation processes. For instance, real-time sensors and monitoring systems can track variables such as temperature, pH, oxygen levels, and microbial growth rates [207]. These systems are increasingly integrated with ML algorithms, which can analyze large datasets in real-time and predict optimal conditions for maximizing yield. ML can also adjust fermentation parameters dynamically based on real-time data, improving process stability and ensuring a consistent production of high-quality polymer precursors [208]. Figure 3 is a visual representation of the key factors influencing fermentation in biopolymer production.

### 2.3. Polymerization and Extraction

After the production of polymer precursors via fermentation, the next stage in biopolymer manufacturing involves polymerization and extraction. These processes transform the monomeric precursors, such as lactic acid or hydroxyalkanoates, into long-chain polymers that exhibit the desired mechanical and thermal properties [184]. The choice of polymerization method, extraction techniques, and subsequent purification affects the final properties of the biopolymer, as well as the overall sustainability and cost-effectiveness of the manufacturing process [209,210,211].

Polymerization is the chemical process by which monomer molecules are joined to form long-chain polymers [212,213,214]. In biopolymer production, this step can involve different techniques depending on the specific biopolymer being synthesized. The two primary methods for polymerizing biopolymers like polylactic acid (PLA) [215,216,217] and polyhydroxyalkanoates (PHAs) [218,219,220] are chemical polymerization and microbial synthesis, respectively.

In the production of polylactic acids (PLAs), the precursor lactic acid undergoes two main types of polymerization.

Lactic acid monomers are linked together via condensation reactions, during which water is released as a by-product [221]. This method is typically used for creating low-to-medium-molecular-weight PLAs. However, it is often less efficient for achieving high-molecular-weight polymers, which are needed for certain applications that require greater mechanical strength.

To produce higher-quality PLAs, the lactic acid is first converted into lactide, a cyclic dimer, which then undergoes ring-opening polymerization. In this process, a catalyst (often a metal-based catalyst) initiates the opening of the lactide ring, allowing it to polymerize into long PLA chains. ROP is the preferred method for producing high-molecular-weight PLAs due to its ability to create strong, durable polymers with controlled molecular architecture [222,223]. The temperature, catalyst type, and reaction time must be carefully optimized to achieve the desired polymer properties, such as molecular weight, crystallinity, and thermal stability.

Unlike PLAs, polyhydroxyalkanoates (PHAs) are synthesized directly by microorganisms through a biological polymerization process. In nutrient-limited conditions (e.g., limited nitrogen or phosphorus), certain bacterial strains such as Cupriavidus necator or Ralstonia eutropha store carbon and energy in the form of intracellular PHA granules [224]. These granules, consisting of long-chain PHA polymers, are stored inside the bacterial cells as reserve materials. The composition and properties of the PHA polymer can vary depending on the microorganism and the carbon source used in fermentation. For instance, the production of poly(3-hydroxybutyrate) (PHB), the most common PHA, is optimized by adjusting the feedstock and fermentation conditions [225]. After microbial synthesis, the PHA polymer needs to be extracted from the cells through physical or chemical processes.

Once the polymerization stage is complete, the next challenge is to extract the biopolymer from the reaction medium or, in the case of PHAs, from the microbial cells themselves [226]. Extraction techniques vary depending on the biopolymer and production process, but the goals are to isolate the polymer with high purity and ensure that its properties are not degraded during extraction [35].

For PLAs, the polymerized material needs to be separated from the reaction medium and any residual catalyst [227]. This is typically performed using filtration, precipitation, or solvent-based methods, depending on the production scale and the intended use of the PLA. The extracted PLA is then purified to remove any unreacted monomers or by-products. For commercial-scale production, processes such as melt filtration or solvent precipitation are often employed to produce high-purity PLA granules. After purification, the polymer is typically dried to remove any moisture, which can degrade the polymer during storage or further processing [228,229].

For PHAs, the extraction process is more complex because the polymer is stored inside microbial cells [219]. Several methods can be used for extracting PHAs. Organic solvents like chloroform, acetone, or methylene chloride can be used to dissolve the PHA granules from the bacterial cells [230]. This is a widely used method, but it can be energy-intensive and may involve toxic solvents, raising concerns about environmental safety and waste disposal. Another method involves the mechanical disruption of the bacterial cells, using techniques such as high-pressure homogenization, sonication, or bead milling, to physically break open the cells and release the PHA granules [35,231,232]. Once released, the PHA is recovered using filtration or centrifugation. More environmentally friendly methods involve enzymatic degradation of the non-PHA cellular components [233], or aqueous-based extraction [234], where the cell material is solubilized in water under specific conditions, leaving the PHA granules intact. These methods are still being optimized for industrial-scale applications but offer the advantage of reducing the need for toxic solvents.

Once extracted, the biopolymers undergo purification to remove impurities, unreacted monomers, residual solvents, and other by-products that could affect their performance or safety [26]. The specific purification methods depend on the type of polymer and the extraction process used. For example, PLA purification often involves solvent evaporation or crystallization techniques, while PHA purification may require additional washing and drying steps to remove residual bacterial cell fragments or solvents [235].

The purified polymers may then undergo post-processing to tailor their physical properties for specific applications [236]. This can include adjusting their molecular weight, crystallinity, or blending with other additives to enhance properties such as flexibility, impact resistance, or thermal stability. For instance, PLA might be blended with plasticizers to improve its flexibility for use in packaging, while PHAs might be blended with other biopolymers to enhance their durability for use in medical devices or agricultural films [237,238].

The extraction and purification steps in biopolymer production are often energy-intensive, involving processes like solvent evaporation, filtration, and drying, which require energy inputs [239]. For example, Kavitake et al. [240] focused on the extraction, purification, and characterization of an exopolysaccharide (EPS) from *Enterococcus hirae* OL616073, a strain isolated from Indian fermented food. The EPS was purified using ion exchange and size exclusion chromatography, yielding two major fractions with molecular masses of 7.7 × 10^4^ and 6.5 × 10^4^ Da. Structural analysis by FTIR, HPTLC, GC-MS, and NMR revealed that the EPS is a homopolysaccharide composed of glucose with α-(1 → 6) and α-(1 → 3) glycosidic linkages. The EPS demonstrated excellent physico-functional properties, including high water solubility, oil holding capacity, emulsifying activity, and shear-thinning rheology. These findings suggest that this EPS could be a promising functional biopolymer for applications in the food and pharmaceutical industries. If not carefully managed, these processes can offset the environmental benefits of using renewable feedstocks and biodegradable materials. The use of organic solvents in extraction, in particular, poses environmental challenges due to the potential for solvent emissions, hazardous waste, and energy consumption in solvent recovery or disposal.

**Figure 4:**
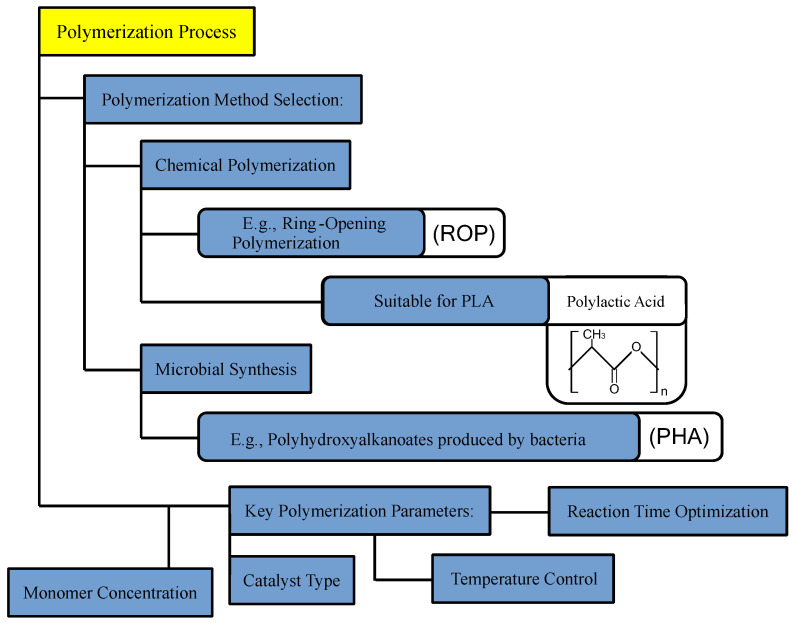
Stages and key steps involved in polymerization and extraction during biopolymer production.

To ensure that the environmental advantages of biopolymers are maintained, manufacturers are focusing on developing more energy-efficient and environmentally friendly extraction methods. For example, there is growing interest in using supercritical CO_2_ extraction as an alternative to organic solvents [241,242]. Supercritical CO_2_ is a non-toxic, non-flammable solvent that can be used at relatively low temperatures, reducing energy consumption and eliminating hazardous solvent waste [243]. The extraction of rare and precious metals from waste is becoming essential as mineral resources deplete, with supercritical CO_2_ extraction emerging as a promising, eco-friendly solution. Supercritical CO_2_ has unique properties, such as liquid-like solubility and gas-like mass transfer, allowing it to penetrate porous materials and extract metals effectively [244]. This method can be optimized by adjusting temperature and pressure to selectively dissolve and recover target metals while reducing unwanted by-products and solvent waste. Studies demonstrate high efficiency in extracting metals like copper, lead, and rare earth elements using supercritical CO_2_ combined with modifiers. Compared to conventional hydrometallurgical and pyrometallurgical methods, supercritical CO_2_ offers enhanced sustainability and purity. By improving process parameters and integrating advanced techniques, this technology has the potential to revolutionize waste recycling and metal recovery. Similarly, advances in biorefining technologies aim to integrate biopolymer production with other bio-based processes to minimize energy use and maximize resource efficiency [245]. Figure 4 is a diagram illustrating the stages and key steps involved in polymerization and extraction during biopolymer production.

### 2.4. Quality Control and Post-Processing

After the polymerization stage, biopolymers undergo quality control to ensure that they meet the desired specifications for mechanical strength, thermal stability, and biodegradability [55,246]. Any inconsistencies in the polymer’s properties—such as molecular weight, crystallinity, or impurity levels—can affect the final product’s usability, durability, and biodegradability [247]. Quality control typically involves testing for mechanical properties, thermal stability, molecular weight, purity, and crystallinity. For example, mechanical strength is tested through tensile tests to ensure that the material can withstand the required stress for applications like packaging or automotive components [248,249]. Thermal stability, tested using differential scanning calorimetry (DSC) or thermogravimetric analysis (TGA), ensures that the polymer can endure temperature variations without degrading [250,251]. Molecular weight and purity are evaluated using techniques like gel permeation chromatography (GPC) or nuclear magnetic resonance (NMR), while crystallinity, which affects the polymer’s flexibility and transparency, is assessed using X-ray diffraction (XRD) or scanning electron microscopy (SEM) [252].

Biodegradability is a defining feature of biopolymers, and thorough testing ensures compliance with environmental standards such as ASTM D6400 [253,254,255] or EN 13432 [256,257]. These tests simulate industrial composting conditions to confirm that the material breaks down within a specified time frame and does not leave toxic residues. Ensuring biodegradability under the right conditions is crucial, especially for products marketed as compostable or environmentally friendly.

After passing quality control, biopolymers often undergo post-processing to enhance their properties for specific applications. Post-processing may include blending the biopolymer with additives such as plasticizers, which improve flexibility [258], or UV stabilizers [259], which protects the material from degradation when exposed to sunlight. For example, PLA is often brittle, so it is commonly blended with plasticizers like glycerol or oligomers to make it more flexible for use in packaging films [238]. PHAs, which are more thermally stable, might require impact modifiers to improve their toughness for applications like medical devices or textiles.

In addition to additives, biopolymers can be blended with other polymers, including both biopolymers and conventional plastics, to optimize their properties [209]. For example, blending a PLA with polycaprolactone (PCL) or polybutylene adipate-co-terephthalate (PBAT) improves its flexibility and biodegradability [260,261]. Surface treatments, such as plasma treatment or corona discharge, may be applied to enhance adhesion, printability, or barrier performance, making the material more suitable for packaging or medical applications. In medical contexts, surface treatments can also enhance biocompatibility, ensuring that the polymer is safe for use in implants or drug delivery systems.

Thermal and mechanical post-processing methods are also employed to further modify the polymer’s properties. Thermal treatments, such as annealing, can increase crystallinity in polymers like PLA, improving heat resistance and mechanical strength [262]. Techniques like extrusion, injection molding, and 3D printing are used to shape biopolymers into final products. During these processes, temperature and processing conditions must be carefully controlled to prevent polymer degradation. For instance, in injection molding, the temperature needs to be optimized to avoid thermal degradation, while blown film extrusion is often used for creating biodegradable packaging films [263].

Fungal pathogens are a significant threat to agricultural crops, reducing both the quantity and quality of yields. Usmanova et al. [264] developed innovative seed-coating formulations using biopolymers [209,265], such as polyhydroxyalkanoate (PHA) and pullulan, along with beneficial microorganisms for enhanced plant protection. The microbial strains used (e.g., *Pseudomonas flavescens* and *Bacillus aerophilus*) demonstrated key agricultural properties, including phytohormone production, antifungal activity, and salt tolerance. Pullulan, synthesized by *Aureobasidium pullulans C7*, exhibited ideal viscosity characteristics for seed coating, transitioning from Newtonian to pseudoplastic behavior at higher concentrations. Seed coatings combining microbial inoculants and polymers improved barley growth under phytopathogenic stress, enhancing germination rates, root and stem lengths, and photosynthetic pigment content. This approach highlights the potential of biopolymers and microbial strains to mitigate crop losses and promote sustainable agriculture.

Lastly, ensuring the environmental performance of biopolymers through biodegradability testing and regulatory compliance is crucial. Products must pass standardized tests, such as ISO 17088 [254,256,266] or ASTM D6400 [267,268,269], to ensure they decompose safely in industrial composting environments. These tests assess the rate of degradation, environmental safety, and the material’s ability to disintegrate under specific conditions. The goal is to confirm that biopolymers do not leave harmful residues and degrade within the expected time frames, reinforcing their role as sustainable alternatives to petroleum-based plastics.

The complexity and variability of raw materials, combined with fluctuating environmental and operational conditions, require a robust system for process monitoring and optimization. Traditional process control methods may struggle to keep up with these dynamic factors, leading to inconsistencies in product quality or inefficiencies in production. ML techniques can analyze vast amounts of data generated throughout the production process, enabling real-time monitoring and control.

For example, ML algorithms can be trained to predict the outcomes of fermentation based on the feedstock’s composition, environmental conditions, and microorganism strains, allowing for more precise control of the process [270,271]. Similarly, ML-driven predictive models can forecast fluctuations in raw material quality or identify optimal processing parameters for polymerization and extraction, ensuring consistent quality and minimizing waste [272,273]. Additionally, ML can enable the development of smart manufacturing systems that automatically adjust processing conditions in real-time to maximize efficiency and product performance, reducing the need for manual intervention.

In the realm of quality control, ML-powered systems can detect defects or inconsistencies in the biopolymer’s properties early in the production process, allowing for timely corrections before the final product is manufactured [274]. This can improve yield and reduce resource consumption, making the entire production process more sustainable. Moreover, ML can assist in optimizing the supply chain by predicting demand, optimizing inventory management, and minimizing transportation costs, further enhancing the sustainability of biopolymer manufacturing.

Biopolymer-bound soil composites (BSCs) are innovative, cement-free construction materials utilizing binders like starch, protein, and lignin. While they offer sustainable alternatives for diverse applications [275], their production presents challenges such as internal defects, improper mixing, and compaction issues. Traditional quality control methods, like vision or acoustic techniques, are often inefficient, as they focus on isolated issues and cannot monitor the unique strength gain process during desiccation. To address these gaps, the BioSys system, suggested by Miao et al. [276], employs vibration-based, non-destructive testing to evaluate BSC quality through impulse hammer-generated signals and accelerometer-recorded responses. BioSys utilizes ML models, achieving up to 99% accuracy in detecting defects, 100% accuracy in identifying improper compaction, and a 5% mean absolute percentage error (MAPE) in predicting strength gain. This system’s ability to simultaneously detect multiple defects, monitor compaction, and assess desiccation makes it a powerful tool for scaling the sustainable production of high-quality BSC materials.

**Figure 5:**
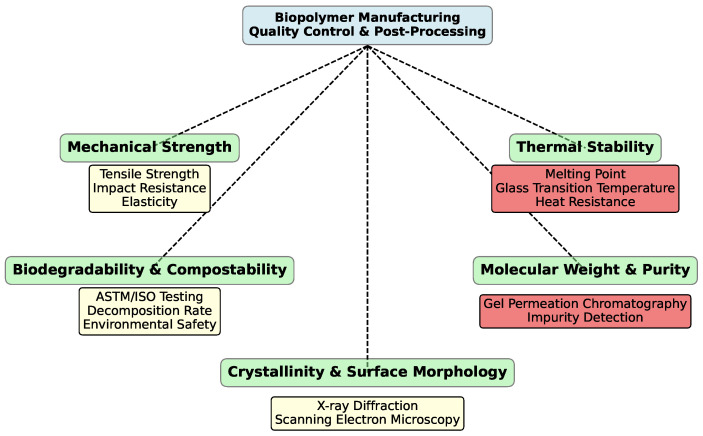
Quality control during biopolymer production.

Figure 5 is a diagram illustrating the stages and key steps involved in quality control during biopolymer production.

## 3. ML Techniques in Process Optimization

The integration of ML techniques in process optimization offers transformative potential for the manufacturing industry, particularly in the realm of biopolymer production. ML-driven tools enable a more precise control over production parameters, real-time adjustments, and better quality assurance. Below, we explore several ML methodologies that are driving innovations in this field.

ML is extensively utilized in process industries due to its ability to analyze vast datasets and make data-driven predictions. For biopolymer manufacturing, ML can be used for monitoring fermentation conditions, predicting product yields, and optimizing complex multi-step processes like extraction and purification [277]. The ability of ML models to continuously learn from production data makes them a vital tool for improving efficiency, minimizing waste, and ensuring consistent product quality [278].

### 3.1. Supervised Learning

**Figure 6:**
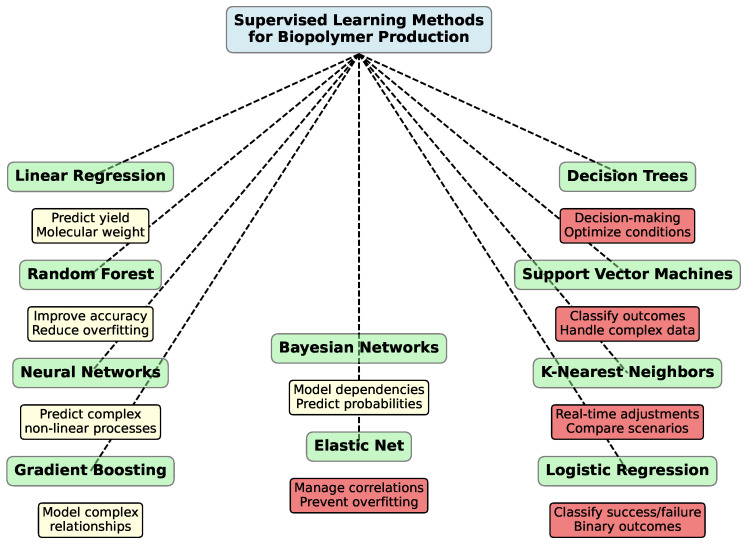
Overview of supervised learning methods applied in biopolymer production.

Supervised learning [279,280,281,282] is particularly useful in biopolymer production because it leverages labeled datasets to train models that can predict future outcomes. Figure 6 presents key supervised learning methods that assist in various aspects of biopolymer production, including predicting yields, molecular weight, and other characteristics of the final product.

In biopolymer processes, this technique can forecast fermentation yields, molecular weight distribution, or viscosity of the final product based on key input parameters such as temperature, pH, feedstock composition, or microbial strain. For instance, predictive models trained on historical data can be employed to recommend adjustments to fermentation conditions in real-time, ensuring optimal performance under varying environmental conditions.

Moman et al. [283] addressed the computational prediction of ligand–biopolymer affinities, emphasizing ML’s role in modern drug discovery. Their work proposes using a nonparametric model of effective radii of atom descriptors, computable for the entire Periodic Table, which, when integrated with MLalgorithms, provides competitive predictive performance. The research involved querying the Protein Data Bank (PDB) [284,285,286] for relevant protein–ligand structures, converting affinity data into a usable format, and cleaning PDB files through automated scripts. The dataset was split into training (60%), validation (20%), and testing (20%) sets across multiple random splits to enhance robustness. ML models, specifically RandomForestClassifier [287,288] and RandomForestRegressor [289,290] from Scikit-learn, were utilized for classification and regression tasks. The final structure–activity database comprises 4703 biopolymer–ligand complexes, forming a valuable resource for predicting ligand affinities.

Biodegradable starch films are promising options for food packaging. Kathuria et al. [291] suggested using the k-Nearest Neighbor [292,293] (KNN) algorithm to classify these films based on parameters such as thickness, water vapor permeability (WVP), tensile strength (TS), and transparency. Twelve films from various botanical starch sources were produced via the casting method, resulting in a database of thirty-six samples. The 5% cassava starch formulation emerged as the best, with WVP 1.21 × 10^−10^ g · m^−1^ · s^−1^ · Pa^−1^, TS 2.34 MPa, thickness 0.193 mm, and water activity (Aw) 0.408. The KNN and principal component analysis effectively classified and selected optimal biodegradable starch films.

The automotive industry seeks cost-effective, renewable materials. Bejagam et al. [294] explored the use of wheat straw as a filler in polypropylene for automotive applications, aiming to meet mechanical property standards set by conventional fillers like glass fiber. Biocomposites [295,296,297] were created by varying the weight percentages of wheat straw and polypropylene through extrusion. The molded products underwent mechanical testing. Predictive models for the biocomposite properties were developed using Polynomial Regression, ANNs, and Support Vector Machines (SVMs). Results indicated that SVMs yielded the best predictive model, followed by ANNs and polynomial regression.

Xing et al. [298] compared an ANN [299,300] and an SVM [301,302,303] for predicting the molecular weight of polycaprolactone (PCL) synthesized via enzymatic catalysis. The study optimized synthesis parameters using a D-optimal design and employed ML techniques to predict the output molecular weight of biopolymers. The biocomposites were created by varying the weight percentages of ϵ-caprolactone and toluene, with mechanical testing performed on the molded products. Both the ANN and SVM were evaluated for prediction accuracy and the SVM was revealed to be the superior method in this context. Experimental data collection involved temperature, time, monomer/solvent ratios, and mixing speed, demonstrating the SVM’s effectiveness in handling the polymerization problem’s characteristics.

Pullulan is a biodegradable hydrogel biopolymer with applications in food, medicine, and cosmetics. Saber et al. [304] utilized the endophytic fungus *Aureobasidium pullulans* (accession number OP924554) for pullulan biosynthesis [305,306,307]. The fermentation process was optimized using Taguchi’s approach [308,309,310] and a decision tree [311,312,313] learning algorithm, which identified key variables affecting pullulan production. The decision tree model successfully reduced sucrose content by 33% without compromising pullulan yield. Optimal nutritional conditions were established as sucrose (60 or 40 g/L), K_2_HPO_4_ (6.0 g/L), NaCl (1.5 g/L), MgSO_4_ (0.3 g/L), and yeast extract (1.0 g/L) at pH 5.5, with a short incubation time of 48 h, achieving a pullulan yield of 7.23%. The structure of pullulan was confirmed through FT-IR [314,315,316] and H1-NMR [317,318,319] spectroscopy. This study marked the first application of Taguchi and decision tree methodologies for optimizing pullulan production, paving the way for further research on using ML to enhance fermentation processes.

Berger et al. [320] evaluated the conversion of orange peels into biodegradable polymers using a decision tree method to identify optimal production variables. The study analyzed factors such as the particle size of orange peel powder, starch types, cooling methods, and dehydration processes. The decision tree approach allowed for the efficient organization and analysis of these variables, leading to the identification of optimal conditions: a particle size of 250 μm, a 100% corn starch ratio, cooling at room temperature, and effective dehydration. The use of a decision tree model facilitated a structured exploration of the best combinations of ingredients and methods for producing high-quality bioplastics [321], demonstrating its effectiveness in optimizing the biopolymer production process.

The depletion of fossil fuels and rising plastic pollution necessitate sustainable alternatives like polyhydroxyalkanoates (PHAs). Bejagam et al. [294] employed ML to predict the melting temperature (Tm) of various PHA homo- and copolymers using a curated dataset of experimental Tm values, molecular weights, and polydispersity indices. Descriptors of polymer topology and charge/polarity were utilized to develop predictive ML models. This approach, integrated with a glass transition temperature (Tg) prediction model and an evolutionary algorithm, facilitated multiobjective optimization in polymer design.

Patnode et al. [322] developed bioplastic films using soy protein, zein, and plant oil-based monomer (POBM) latexes as sustainable alternatives to petrochemical-based food packaging. By leveraging the film-forming ability of soy protein, the strength of zein, and the plasticizing and hydrophobizing effects of POBM-latexes, strong, flexible, and moisture-resistant bioplastic films, termed proteoposites, were created. ML models with >85% accuracy were used to predict and optimize the bioplastics’ properties, confirming experimental outcomes. These proteoposite films show promise as biodegradable, high-performance packaging materials.

Biopolymer-based soil treatment (BPST) [323,324,325] is gaining traction in sustainable geotechnical engineering due to its low carbon footprint and effective ground improvement properties. Lee et al. [326] employed a decision tree ML model to predict the unconfined compressive strength (UCS) of BPST, achieving a high accuracy of R^2^ > 0.99. Their analysis revealed that biopolymer and water contents were critical factors influencing UCS. The model utilized data from eight published studies on BPST, focusing on various features that affect strength, including biopolymer type, soil type, and water content.

Bohar et al. [327] integrated ML and additive manufacturing to predict and optimize the mechanical strength of FDM-printed PEEK components, critical for aerospace, biomedical, and automotive industries. Key process parameters—infill density, layer height, printing speed, and infill pattern—were analyzed experimentally. Support Vector Regression (SVR) and Random Forest Regression (RFR) models achieved accurate tensile strength predictions, with deviations under 5%. Using a genetic algorithm (GA), the optimized parameters yielded a maximum tensile strength of 66.17 MPa. Microstructural analysis validated the results, demonstrating the potential of ML-driven optimization for high-performance 3D printing.

Ergun et al. [328] explored xanthan gum as a foam material for insulation and packaging, a novel application for this natural biopolymer. Foams were produced using varying ratios of xanthan gum and cellulose fiber in a 5% citric acid medium. Results showed that xanthan gum significantly influenced foam properties, with densities ranging from 49.42 to 172.2 kg/m^3^, compressive moduli from 235.25 to 1257.52 KPa, and flexural moduli from 1939.76 to 12,736.39 KPa. Five ML models were applied to predict material properties, with the generalized regression neural network (R^2^ > 0.97) achieving the best accuracy, optimizing the process while reducing experimental efforts.

Ergun et al. [329] investigated the use of guar gum-based foams for insulation applications, focusing on their properties and predicting them through regression analysis. The foams were produced by mixing guar gum, cellulose, and boric acid in varying proportions and drying the mixture. The resulting foams exhibited desirable properties such as low density, low thermal conductivity, and good mechanical strength. Regression models, including Multiple Linear Regression (MLR) and Gaussian Process Regression (GP), were used to estimate the foam’s density, compression modulus, and thermal conductivity. The GP model achieved high prediction accuracy (R^2^ up to 0.99), indicating that guar gum significantly influenced the foam’s properties.

Lofgren et al. [330] explored the optimization of the AquaSolv omni biorefinery for lignin using Bayesian optimization, an ML technique that enhances sample efficiency and guides data collection. The process links biorefinery conditions, such as hydrothermal pretreatment severity and temperature, with lignin’s structural features, analyzed through 2D nuclear magnetic resonance spectroscopy. By applying Pareto front analysis, the optimal processing conditions were identified to maximize lignin yield and preserve β-O-4 linkages for efficient depolymerization into platform chemicals. The research highlighted ML’s potential in advancing sustainable chemical processing for targeted applications.

Ifran et al. [331] developed an ML model using Gaussian Process Regression to predict nutrient release time from biopolymer-coated controlled-release fertilizers (CRFs). The model incorporates parameters like diffusion coefficient, coating thickness, and size distribution. With an R^2^ of 1 and an RMSE of 0.003, the model accurately predicted nutrient release, helping to optimize CRF performance in precision farming. It can be used to analyze and improve the release profiles of various biopolymer-coated CRFs.

Champa et al. [332] enhanced the mechanical properties of poly[(butylene succinate)-co-adipate] (PBSA) using functionalized single-walled carbon nanotubes (SWCNTs). Different SWCNT loadings were incorporated into PBSA via solution casting and optimized ultrasonication. The nanocomposites showed significant improvements in stiffness due to the superior reinforcing ability of SWCNTs. Four machine learning models—Polynomial Regression, Support Vector Machines, Gradient Boosting, and Artificial Neural Networks—were used to predict mechanical properties such as Young’s modulus, tensile strength, elongation at break, and impact strength, with R^2^ values ranging from 0.69 to 0.99. The study offers a promising approach to modeling and optimizing polymeric nanocomposites for various industrial applications.

For clarity, Table 1 summarizes the results of studies that utilized different supervised learning models for analyzing biopolymers. The table includes information on specific materials, applied models, obtained results, and limitations of the research.

### 3.2. Unsupervised Learning

**Figure 7:**
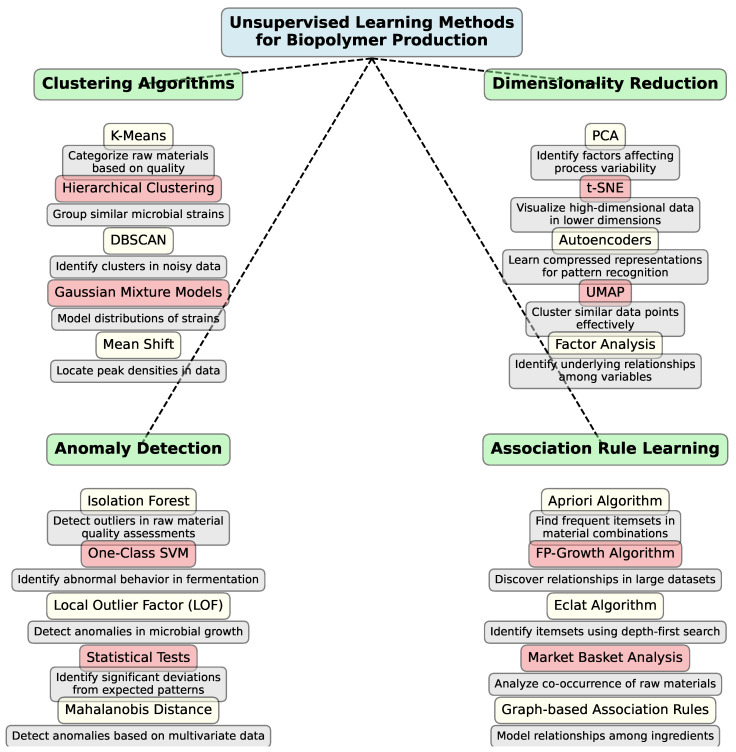
Unsupervised learning methods in biopolymer research with possible applications.

Unsupervised learning is beneficial for exploring and identifying hidden patterns in process data where no predefined labels exist. For example, clustering algorithms can categorize batches of raw materials based on their composition, quality, or suitability for biopolymer production. These techniques are also useful in analyzing microbial behavior during fermentation, where different strains might exhibit unique growth profiles, by grouping them based on similar characteristics or fermentation outcomes. Dimensionality reduction methods like Principal Component Analysis (PCA) can also uncover significant factors contributing to process variability, facilitating better control strategies. Figure 7 illustrates possible ways to apply unsupervised methods in biopolymer research.

Lignin, the second most abundant biological polymer, has a complex structure and is primarily produced as a waste product in the pulp and paper industry, often underutilized. Understanding its structure is crucial for exploring potential applications. High-resolution nuclear magnetic resonance (NMR) spectroscopy is commonly used for dissolved lignin, but it cannot analyze insoluble technical lignins. Solid-state NMR spectroscopy offers a solution. Grishanovich et al. [333] introduced a method to classify the degree of lignin alteration using Hierarchical Cluster Analysis (HCA) on solid-state NMR spectra, addressing the lack of direct correlations between NMR spectra of dissolved and solid lignins.

Ireddy et al. [334] analyzed the surfaces of polyhydroxyalkanoate (PHA) films with varying monomer compositions using atomic force microscopy (AFM) and unsupervised ML algorithms. The aim was to classify films based on global attributes such as scan size, thickness, and monomer type. Their research benchmarked 12 widely used clustering algorithms through a hybrid approach, demonstrating the effectiveness of applying a one-dimensional (1D) Fourier Transform [335] (FT) on high-dimensional vectorized data for classification. Results indicated that the 1D FT produces the most accurate outcomes. The study also provided insights into individual algorithm performances and the impact of different data pools, alongside an early version of a tool designed for surface investigation using ML methods.

PLA is a bioresorbable polymer used in medical devices that require careful processing to avoid degradation. Mulrennan et al. [336] integrated in-process temperature, pressure, and NIR spectroscopy measurements with multivariate regression methods to predict the mechanical strength of extruded PLA products. Their work evaluated the feasibility of this method as an intelligent sensor for real-time quality analysis in compliance with medical device regulations. Their results indicated that combining NIR and conventional sensor data is essential for robust predictions across varying processing conditions. While partial least squares [337] (PLS) performed well, Random Forest (RF) and Support Vector Regression [338] (SVR) demonstrated superior reliability with a prior principal component dimension reduction step, suggesting that nonlinear methods may outperform traditional linear methods in predicting mechanical properties from complex sensor data.

DNA-binding proteins are crucial for genetic information processing but are often inefficiently identified by traditional methods. Zhang et al. [339] leveraged ML to extract and optimize four feature types: Reduced sequence and index-vectors (RS), Pseudo-amino acid components (PseAACS), Position-specific scoring matrix-Auto Cross Covariance Transform (PSSM-ACCT), and Position-specific scoring matrix-Discrete Wavelet Transform (PSSM-DWT). Using the LASSO method for dimension reduction, the optimized features were input into ensemble learning algorithms, achieving high accuracy rates of 86.98% and 88.9% in five-fold cross-validation with datasets PDB1075 and PDB594. The independent experiment showed an accuracy of 83.33%, indicating that the proposed methodology effectively predicts DNA-binding proteins.

The Kohonen self-organizing map [340] (SOM) was utilized by Qiao et al. [341] to map protein molecular surfaces, representing properties like shape and molecular electrostatics through 3D surface coordinates. This approach allows for visual comparisons of molecular features among proteins with similar topological or chemical characteristics. The SOM organizes input features onto a layered NN, creating globally ordered maps while preserving topological relationships and reducing dimensionality. The competitive learning process adjusts weights in the SOM, ensuring that neurons close in the network activate each other based on similar input, leading to global organization. This innovative method addresses the challenges of representing complex interrelationships in computational chemistry and biochemistry.

The consensus scaffolded mixture (CSM) position weight matrix model enhances the modeling of cis-regulatory elements by using overlapping components represented by multiple-position weight matrices (PWMs) linked to specific binding patterns. Jiang et al. [342] introduced a learning algorithm consisting of an initial structure learning phase based on frequent pattern mining, followed by refinement using the expectation maximization (EM) algorithm. In a case study of the transcription factor Leu3, CSM models aligned with conventional mixtures but demonstrated superior fitness via the Fermi-Dirac distribution. An analysis of predicted binding sites for 83 JASPAR transcription factors indicated that the CSM outperformed simple mixtures, context-specific independent (CSI) mixtures, and single PWM models in 83%, 84%, and 75% of the cases, respectively. A five-fold cross-validation across 46 TRANSFAC datasets confirmed the CSM model’s greater generality compared to other mixture models.

Motif discovery [343] in biological sequences is essential for understanding gene expression and regulation. Hasan et al. [344] reviewed the application of data mining techniques for motif discovery, noting a recent surge in interest despite limited prior usage compared to other algorithms. Various methodologies, including GYM, a program based on the a priori method [345], successfully identified helix-turn-helix motifs, improving detection rates without increasing false predictions. Challenges included the choice of training sets and minimum support thresholds. The modified prefix span method improved frequent pattern extraction by considering gaps, while BioPM utilized a prefix-projected method for efficient motif mining. Pushdown automata were employed for grammar-based motif extraction, and algorithms like informative motif mining and FP-growth enhanced performance by optimizing the search for biologically significant motifs.

Yousef et al. [346] explored how learning a suitable distance metric from labeled examples can significantly enhance k-Nearest Neighbor (kNN) classification performance. The proposed ensemble clustering kNN classifier [347] (EC-kNN) improved accuracy by defining distances based on co-clustering rather than solely geometric proximity. Through experiments involving seven plant microRNA species and eight feature selection methods, EC-kNN consistently outperformed traditional classifiers, including SVM. The EC-kNN approach also reduced data complexity by grouping points into equivalence classes, facilitating a novel data reduction technique complementary to methods like principal component analysis (PCA). The algorithm’s effectiveness was demonstrated through multiple runs and robust average results across different datasets.

Wei et al. [348] addressed the challenge of limited data in biochemistry, particularly in organic chemistry. To enhance modeling performance in the biopolymerization process, the authors proposed an ML approach that utilizes variational autoencoders and generative adversarial networks for data augmentation, mitigating overfitting. The Random Forest and ANN algorithms were employed for modeling. Results indicated that data augmentation significantly improves regression model performance, with the Random Forest model augmented by generative adversarial networks achieving the highest predictive accuracy—an R^2^ of 0.94 on the training set and 0.74 on the test set.

Lignin, an abundant biopolymer, presents substantial industrial potential, yet the limited molecular structure data restrict its applications. Eswaran et al. [349] introduced the Lignin Structural (LGS) Dataset, which features the molecular structures of milled wood lignin, emphasizing on two primary monomeric units (coniferyl and syringyl) and six prevalent interunit linkages. The dataset encompasses 60,000 newly generated lignin structures that accurately reflect experimental properties, achieving about 90% accuracy in matching literature data. The LGS dataset serves as a crucial resource for advancing lignin chemistry research, supporting computational simulations and predictive modeling.

Abreu et al. [350] investigated biohydrogen production from arabinose using four different anaerobic sludges across varying pH levels (4.5 to 8.0), with arabinose concentrations set at 30 g/L. The modified Gompertz equation was used to estimate production parameters, revealing that higher pH values led to greater hydrogen production across all sludges. Among the tested sludges, G2 (acclimated granular sludge) demonstrated the highest hydrogen yield and arabinose consumption. Granular sludges exhibited distinct behavior from suspended sludges, including shorter lag phases and varying fermentation pathways. A strong correlation (R^2^ = 0.973) between n-butyrate and ethanol percentages in G1 sludge suggested that ethanol/butyrate fermentation was predominant, while S1 showed a high correlation between n-butyrate and acetate (R^2^ = 0.980). The findings imply that granular sludge maintains efficiency across broader pH ranges, optimizing the hydrogen production of arabinose.

Fredricks et al. [351] highlighted the environmental concerns associated with non-degradable fossil-based plastics and advocates for biopolymers as sustainable alternatives. Biopolymers, synthesized by living organisms, offer desirable mechanical properties, compostability, and renewable sourcing. The paper discusses the hierarchical structure of three prominent biopolymer classes—cellulose, chitin, and protein beta-sheet structures—focusing on how their interaction networks contribute to mechanical strength. Various fabrication and processing techniques to develop macroscopic materials and composites from these biopolymers were reviewed. In addition, a novel approach that uses intact microorganisms or biological matter as building blocks for material construction was presented. The paper emphasizes the processing–structure–property relationships of biomatter-based materials and concludes with a perspective on the potential role of biopolymers in promoting a circular economy.

To provide a clear overview of key research in the fields of biopolymers and unsupervised learning, Table 2 summarizes the main studies. It highlights the research focus, materials used, applied models, results, and identified limitations.This summary facilitates a comparison between different approaches and models used in biopolymer analysis and related areas.

### 3.3. Neural Networks and Deep Learning

**Figure 8:**
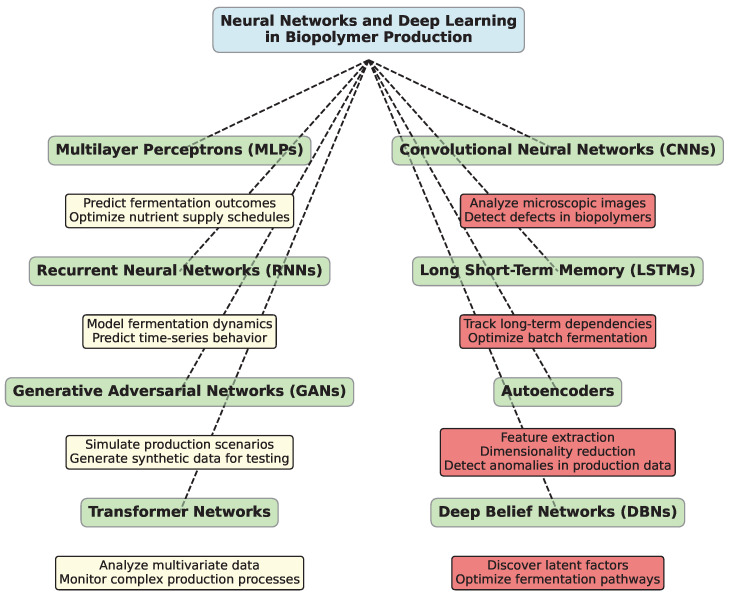
Application of various NNs and deep learning architectures in biopolymer production.

NNs, and more specifically deep learning architectures, handle highly complex, nonlinear relationships between variables within biopolymer production processes. These models excel in situations where traditional statistical models may fall short due to the sheer complexity of interactions, such as those observed in fermentation and polymer extraction. Deep learning can account for numerous variables and their interdependencies, enabling better control and optimization across different stages of the production pipeline. Figure 8 illustrates diagram with key NN architectures used in biopolymer production and their specific applications.

Multilayer perceptrons [352,353,354,355] (MLPs) are a type of NN capable of approximating functions and making accurate predictions based on multiple input variables. In biopolymer manufacturing, MLPs can be used to predict fermentation outcomes based on real-time sensor data, such as pH, dissolved oxygen, and nutrient concentrations. These models can also optimize nutrient supply schedules to maximize microbial activity, thereby enhancing product yield and quality. Additionally, MLPs may assist in adaptive control systems that automatically adjust fermentation parameters during the process, leading to increased production efficiency.

Convolutional Neural Networks [356,357,358,359] (CNNs), though traditionally associated with image recognition, are finding innovative applications in biopolymer manufacturing, particularly in quality control. By analyzing microscopic images of biopolymers, CNNs can detect structural inconsistencies, contamination, or defects that may not be visible through conventional inspection methods. Furthermore, CNN-based systems could be employed in automated defect detection during the final stages of product refinement, ensuring only high-quality biopolymers reach the end users.

Recurrent Neural Networks [360,361,362,363] (RNNs) are specifically designed to handle sequential data, making them highly valuable for time-series prediction. In biopolymer production, RNNs can be employed to model fermentation dynamics by analyzing historical data from previous batches and forecasting future states. This capability enables real-time adjustments in fermentation parameters, reducing the likelihood of deviations and improving consistency in product quality.

Long short-term memory [364,365,366,367] (LSTM) networks are a specialized type of RNN designed to overcome the limitations of short-term memory in traditional RNNs. In biopolymer production, LSTMs can be applied to track long-term dependencies in complex processes, such as the progression of fermentation over extended periods. LSTMs are particularly useful in monitoring and predicting batch fermentation outcomes, optimizing nutrient input, and ensuring the stability of production processes over time.

Generative adversarial networks [368,369,370,371] (GANs) consist of two NNs (a generator and a discriminator) that work in opposition to improve the performance of both. In biopolymer production, GANs can be used to simulate the effects of different production parameters on product yield, aiding in process optimization [372,373]. Additionally, GANs can generate synthetic datasets that replicate the conditions of rare or expensive experiments, helping manufacturers explore different scenarios without conducting costly physical tests.

Autoencoders [374,375,376,377] are unsupervised learning architectures used for feature extraction and dimensionality reduction. In biopolymer manufacturing, autoencoders can be applied to compress large sets of sensor data collected during fermentation and extraction processes. This allows for a more efficient analysis of underlying patterns, leading to better control of key production variables. Autoencoders are also useful for anomaly detection, identifying irregularities in the data that could indicate process faults or contamination.

Transformer networks [378,379,380,381], originally developed for natural language processing, are gaining traction in industries requiring the analysis of long-range dependencies. In biopolymer production, transformers could be used to analyze multivariate time-series data, such as environmental conditions or equipment sensor data, and predict future states of the fermentation process. Their ability to handle large datasets and model complex relationships makes them highly applicable in optimizing batch production cycles.

Finally, Deep Belief Networks [382,383,384,385] (DBNs) are generative NNs that stack multiple layers of Restricted Boltzmann Machines (RBMs). They can learn to represent data hierarchically, making them useful for modeling complex relationships between variables in biopolymer production. DBNs can be applied to tasks such as optimizing fermentation pathways by discovering latent factors influencing microbial growth, leading to more efficient and controlled production.

Biological systems inspire materials science through their complex multiscale architectures. Combining ML with multiscale modeling provides insights into the structure–property–function relationships of biomaterials. Arevalo et al. [386] reviewed ML techniques—such as NNs and autoencoders—that are applied to predict and design biological materials, advocating for the integration of ML with physics-based models for high-throughput materials discovery.

Khare et al. [387] applied transformer models to predict the thermal stability of collagen triple helices based on amino acid sequences. They compared a small transformer model and a large pretrained ProtBERT model. Despite ProtBERT’s higher complexity, the small model achieved a nearly similar accuracy while using significantly fewer parameters. Both models showed good performance against experimental data, marking the first use of transformers for predicting biophysical properties from small data sets.

Bandyopadhyay et al. [388] presented a method to explore the conformational landscapes of mini-proteins and peptides using autoencoders. By projecting molecular dynamics simulations into a latent space, the method identifies key metastable states and predicts the folding behavior of complex proteins. The approach outperformed traditional dimensionality reduction techniques, offering a more optimized view of protein dynamics and folding pathways.

A generative model based on variational autoencoders was developed by Sadeghi et al. [389] to design DNA-stabilized silver nanoclusters (AgN-DNAs) with optimized fluorescence properties. This model allows for multiobjective property design, including the ability to generate AgN-DNAs with enhanced near-infrared emission for bioimaging. It also provides automatic feature extraction and reverse mapping from desired properties to DNA sequences, improving upon traditional models that require manual feature engineering.

Satteri et al.’s [390] review covers recent advancements in data-driven approaches for the inverse design of polymers with specific properties. It highlights three key strategies, all of which leverage materials data to explore chemical space efficiently: high-throughput virtual screening, global optimization, and generative models. The article discusses the challenges and opportunities in using these data-driven techniques to optimize polymer design.

ML techniques were applied by Baldizon et al. [391] to improve the classification of linear and circular DNA molecules in noisy data from solid-state nanopore experiments. Three methods—k-means clustering, principal component analysis with k-means, and long short-term memory (LSTM) models—were tested, with the LSTM model achieving the highest accuracy (80%), demonstrating its potential for better handling of noisy nanopore data.

Noor et al. [392] applied NNs, enhanced by bootstrap resampling, to predict the molecular weight of biopolymers produced in a batch reactor. The biopolymerization process, catalyzed by *Candida antarctica* lipase B, involved ε-caprolactone and toluene. NNs with a single hidden layer and trained with Levenberg–Marquardt optimization were used to model the process, using reaction temperature, time, and molecular weight as inputs. The model achieved accurate one-step-ahead predictions of biopolymer molecular weight, demonstrating its potential for controlling biopolymer quality.

Leal et al. [393] detailed the creation of a hydroxypropyl cellulose (HPC)-based sensor for estimating force. By mixing HPC with deionized water at varying concentrations, the sensor’s RGB color responses were analyzed, showing a correlation between HPC concentration and sensor sensitivity. A 63% HPC concentration yielded the highest sensitivity for red and green components, while a 57% concentration showed uniformity in sensitivity when force was applied at different positions. The sensor demonstrated sub-centimeter spatial resolution for force distribution assessment, and the integration of a CNN improved accuracy, achieving a mean squared error of 0.037.

Salma et al. [394] focused on predicting the drug release and skin permeation of Piroxicam (PX) topical films made from chitosan (CTS), xanthan gum (XG), and their carboxymethyl derivatives (CMXs). Using the solvent casting method with Tween 80 as a permeation enhancer, the films showed good physicochemical properties. Deep learning and ML models were employed to predict drug release and permeation rates. The optimal formulation (F8 based on CTS-CMX3) achieved a 99.97% drug release. The Deep Neural Network (DNN) emerged as the best predictive approach, demonstrating high accuracy with mean squared error values of 0.00098 for drug release and 0.00182 for permeation kinetics.

Araujo et al. [395] utilized thermogravimetric analysis to investigate chitosan’s thermal degradation under dynamic conditions, employing a multilayer perceptron (MLP) NN to quantify contributions from various kinetic models. The MLP architecture successfully approximated experimental data, showing the lowest residual error and determining activation energies ranging from 98.1 to 183.3 kJ/mol. The analysis revealed a relationship between activation energy increases and polymer dehydration, highlighting the MLP’s ability to capture complex thermal behavior during chitosan decomposition.

Wong et al. [396] discussed the biopolymerization of ε-caprolactone using the Novozyme 435 catalyst, varying reactor temperatures and impeller speeds. A multilayer feedforward neural network (FFNN) model was developed, comparing the performance of 11 training algorithms. Results indicated that the quasi-Newton and Levenberg–Marquardt algorithms outperformed others, achieving mean absolute percentage error (MAPE) values of 4.512%, 5.31%, and 3.21% for various molecular weight measures in the polycaprolactone biopolymerization process. This research identified effective training methods for estimating biopolymerization performance.

Laycock et al. [397] discussed the transition from traditional experimental methods to advanced computational approaches in the design and manufacture of biodegradable and bioderived polymeric materials. The Materials 4.0 framework integrates multiscale simulations, computational modeling, and artificial intelligence to model biopolymer structures, predict properties, and understand flow and processability. This holistic approach complements experimental techniques, facilitating the study of various biopolymeric materials, including biodegradable polyesters and polysaccharides. Furthermore, ML techniques were applied to optimize material properties and predict the effects of modifications and external factors. The article emphasizes the growing repository of computational modeling data that enhance design flexibility and processing options before costly experimental testing.

Kartal et al. [398] focused on the thermal degradation of biopolymeric structures in biomass—specifically hemicellulose, cellulose, and lignin. Given the complex structure of biomass, characterizing thermal degradation typically requires extensive experimental resources. The authors developed an ANN model to generate differential thermogravimetric analysis (DTG) curves for these biopolymers using proximate analysis results. Implemented with TensorFlow, the ANN model demonstrated excellent performance with R^2^ values exceeding 0.998, allowing for the estimation of thermal degradation at any temperature. This model enables immediate calculations of biopolymer fractions in degraded biomass, representing a novel advancement in the field.

Review [152] highlights the integration of ML with algae-derived biopolymers for enhancing 3D printing processes. It addresses the need for sustainable manufacturing solutions and discusses algae-based biopolymers like alginate and carrageenan, emphasizing their environmental advantages and technical challenges. The paper outlines how ML can optimize material selection, predictive modeling, and quality control, resulting in improved mechanical properties and printing parameter optimization [399,400,401]. Applications, such as Spirulina-based materials and carrageenan in bone tissue engineering, are highlighted. The article concludes that despite challenges, combining ML with algae-derived biopolymers has the potential to revolutionize sustainable additive manufacturing, with significant advancements in eco-friendly production techniques.

Asgharzadeh et al. [402] presented a deep learning method for segmenting biopolymer networks observed through confocal laser scanning microscopy (CLSM). The authors utilized an encoder–decoder network architecture, achieving a dice score of 0.88 for segmenting filamentous temperature-sensitive Z proteins from the chloroplasts of Physcomitrella patens. The segmentation process involved creating ground truth images through a semi-automated method, using adaptive local thresholding followed by expert modification. To enhance the dataset, 3D images were transformed into 2D slices, resulting in a training dataset of 15,015 images. The model was trained using a 5-fold cross-validation scheme, and performance was evaluated using the Intersection-over-Union (IoU) metric. The network, implemented in Keras and trained on an Nvidia GTX 1070 GPU, successfully produced segmented 3D images from the original CLSM data.

Leng et al. [403] discussed the development of an artificial fully connected neural network (FCNN) for modeling the behavior of representative volume elements (RVEs) in biopolymer gels, such as fibrin and collagen, which are important in tissue engineering. The FCNN was trained on data from 1100 fiber networks under biaxial deformations to predict strain energy derivatives. By incorporating constraints like the convexity of the strain energy function and symmetry of the Hessian, the FCNN was successfully integrated into the finite element software Abaqus as a user material subroutine (UMAT). The model outputs derivatives of strain energy in relation to deformation invariants, enhancing the simulation of biopolymer gels in nonlinear elasticity problems. The authors emphasized the potential for combining ML with computational mechanics to improve the modeling of biological materials with multiscale structures.

The growing environmental concerns over plastic pollution have heightened interest in producing biodegradable starch-based films. Nobrega et al. [404] emphasized the need for a comprehensive understanding of how various additives affect the properties of these films. Self-organizing maps (SOMs) were employed to analyze the mechanical and barrier properties of the films, highlighting the critical role of glycerol in films with low amounts of poly(butylene adipate-co-terephthalate) (PBAT) and its dependence on equilibrium relative humidity for water vapor permeability (WVP). The research utilized a multilayer perceptron model combined with a genetic algorithm to predict and optimize the properties of biodegradable films, achieving a high correlation between experimental and theoretical results with a maximum error of 24%. The authors suggested that further data are needed to enhance the model’s accuracy and ensure component compatibility.

Table 3 summarizes various studies that focus on the use of ML in the design and optimization of biopolymers. For each study, the key focus, materials investigated, applied models, results obtained, and limitations of the approaches are highlighted. This table illustrates the broad range of ML applications in materials science, emphasizing both the advancements made and the challenges that remain in this field.

## 4. Challenges of Integrating ML in Biopolymer Research

Integrating ML into biopolymer research presents several challenges that limit its broader application and effectiveness. One of the primary issues is the limited availability of experimental data. In fields like biochemistry and organic chemistry, data collected from experiments often come in small quantities, making it difficult to train and validate robust models. For instance, in the biopolymerization process, small datasets can lead to overfitting, reducing the ability of models to make accurate predictions on new data. This data scarcity was exacerbated during the COVID-19 pandemic, which further restricted the ability to conduct experimental research.

Additionally, the molecular structures of biopolymers are complex and diverse, posing another major hurdle for accurate modeling. Structures such as lignin or polylactides contain various intermolecular interactions and bonds, making it challenging to mathematically represent these materials using conventional methods. Traditional ML models, like linear regression, often struggle to capture the nonlinear dependencies that characterize these systems. Therefore, more sophisticated modeling techniques and their integration with physical and chemical simulation methods are required.

Another key issue is the lack of high-quality labeled data, especially in biochemical processes. Automating the annotation of datasets is a significant effort, and without reliable labels, ML models cannot achieve high accuracy. Below are key comments based on reviewed papers:

- Using variational autoencoders (VAEs) and generative adversarial networks (GANs) to synthesize new data from small experimental datasets can enhance model quality and mitigate the risk of overfitting. This approach has already been proven effective in certain biopolymer studies.
- Applying nonlinear methods such as Random Forests, SVM, and NNs can significantly improve the prediction of biopolymer properties. These algorithms are particularly useful for handling data with complex molecular interactions.
- ML in biopolymer research can benefit from closer integration with traditional computational chemistry methods, like molecular dynamics or quantum chemistry simulations. Combining knowledge from fundamental laws with ML capabilities will enable more accurate predictions.
- Active learning algorithms can efficiently use small datasets by selecting the most informative experiments to prioritize data collection. This strategy can reduce the experimental workload required to train models.
- As demonstrated by the Lignin Structural Dataset (LGS), the creation and publication of unique datasets for different biopolymers is importnant for advancing the field. These databases will support improved simulations, predictive models, and facilitate resource sharing among researchers.

## 5. Future Directions for Development

To successfully integrate ML into biopolymer research, it is essential to address current challenges and explore future development opportunities. In the future, improving the integration of ML into biopolymer research will aid in the development of new materials with targeted properties and optimize their production for sustainable use. These advancements will help overcome current limitations and open new avenues for innovation in biopolymer science.

Uncertainty quantification (UQ) helps account for variability in input data, measurement errors, and process instability [405]. It allows for assessing the accuracy of models and predictions, providing insights into the confidence of results. In biopolymers, UQ can be used to consider factors like composition, production conditions, and environmental influences on material properties. It can improve the prediction of material characteristics, optimize production processes, and increase the reliability of final products.

Explainable AI (XAI) [406] aims to make machine learning models more interpretable by providing insights into how decisions are made. It helps bridge the gap between model complexity and human understanding, making it easier to trust and validate model outputs. In biopolymer manufacturing, XAI can be applied to understand the relationship between raw materials, processing parameters, and the resulting properties of the biopolymer. This transparency can guide decision-making and improve the optimization of production processes.

Below are several key areas where ML can make a significant impact:

- Grishanovich et al. (2024) [333] used Hierarchical Cluster Analysis (HCA) to classify lignin alterations using solid-state NMR spectra, addressing the gap between dissolved and solid lignins. Ireddy et al. (2024) [334] demonstrated that 1D Fourier Transform (FT) achieved high accuracy in classifying polyhydroxyalkanoate (PHA) films using unsupervised machine learning algorithms. Both approaches highlight the effectiveness of unsupervised techniques in classifying complex biopolymers. However, the accuracy of these models is constrained by the limitations of the underlying technologies (solid-state NMR and FT) and may vary with material composition. Future research should focus on integrating more advanced spectroscopic techniques or hybrid models to overcome these limitations and improve generalization across different biopolymer types.
- Mulrennan et al. (2022) [336] combined near-infrared (NIR) and conventional sensor data with Random Forest (RF) and Support Vector Regression (SVR) models to predict the mechanical strength of polylactide (PLA). Similarly, Bejagam et al. (2022) [294] demonstrated that Support Vector Machines (SVMs) excelled in predicting the mechanical properties of wheat straw-filled polypropylene composites. Both studies show the superiority of nonlinear models like RF and SVM over traditional linear methods for material property prediction. However, the complexity of these models and the need for real-time data or specific formulations may limit practical applications. Future work could focus on simplifying these models for broader use and exploring their adaptability to different biopolymer formulations.
- Zhang et al. (2021) [339] used ensemble learning for high-accuracy DNA-binding protein prediction, relying on feature selection with LASSO. Xing et al. (2002) [298] employed an SVM to predict the molecular weight of polycaprolactone (PCL), showing that SVM outperformed Artificial Neural Networks (ANNs) in this context. While these methods provide high accuracy, they require large, high-quality datasets for training and may not generalize well across different protein types or polymers. Future research could explore methods for data augmentation or transfer learning to expand these models’ applicability and robustness.
- Qiao et al. (2001) [341] employed self-organizing maps (SOMs) for visualizing protein molecular surfaces, while Bandyopadhyay et al. (2021) [388] used autoencoders to predict protein dynamics and folding pathways. Both approaches highlight the importance of unsupervised learning in understanding complex biomolecular features. However, the effectiveness of an SOM depends on input feature quality, while autoencoders may struggle with very complex datasets. Future studies could integrate SOMs with deep learning-based feature extraction or enhance autoencoders by incorporating reinforcement learning to better model protein conformational landscapes.
- Sadeghi et al. (2024) [389] used variational autoencoders (VAEs) for multiobjective optimization in the design of DNA-stabilized silver nanoclusters. Satteri et al. (2021) [390] emphasized the potential of data-driven models, such as generative models, for polymer design. Both studies demonstrate the power of data-driven techniques in optimizing material properties, but challenges remain in data quality and model generalizability. Future research should focus on improving model robustness and combining these techniques with traditional methods to achieve more accurate and versatile material design processes.
- Kartal et al. (2023) [398] employed Artificial Neural Networks (ANNs) to predict the thermal degradation of biomass biopolymers with high accuracy, while Khare et al. (2022) [387] demonstrated the potential of small transformer models to predict the thermal stability of collagen triple helices. Both studies underline the importance of accurate prediction of biopolymer degradation, though the complexity of biomass and the limitations of smaller datasets in transformers may pose challenges. Future studies could integrate more advanced models, such as hybrid machine learning techniques, and explore the use of multi-modal datasets to improve prediction accuracy for biopolymer stability and degradation.
- Ifran et al. (2020) [331] used Gaussian Process Regression (GPR) for accurate prediction of nutrient release in biopolymer-coated controlled-release fertilizers (CRFs), while Kathuria et al. (2022) [291] applied k-Nearest Neighbor (k-NN) models to optimize biodegradable starch film formulations. Both approaches show promise in predicting biopolymer properties, but their applicability may be limited by specific material conditions or dataset sizes. Future research should explore expanding these models to include a broader range of materials and applications and work towards integrating them with other predictive models for improved generalization.
- Khare et al. (2022) [387] demonstrated that small transformer models can efficiently predict the thermal stability of biopolymer structures like collagen triple helices. These models provide a promising alternative to larger models such as ProtBERT, offering similar accuracy with fewer parameters. Future research could investigate the scalability of transformer models for larger, more complex datasets and explore their application to other biopolymer stability predictions.
- Wei et al. (2022) [348] emphasized the importance of data augmentation in improving machine learning model performance for biopolymerization modeling. By enhancing the dataset, they were able to significantly boost prediction accuracy. Future research could focus on developing more robust data augmentation techniques and incorporating generative models, such as GANs, to handle real-world data variability and improve prediction reliability in biopolymer-related fields.

## Figures and Tables

**Table 1:** Summary of supervised learning applications in biopolymer research.

Reference	Focus	Material	Applied Model	Results	Limitations
Lee et al. (2023) [326]	Predicting ligand–biopolymer affinities	Biopolymer–ligand complexes	Random Forest Classifier, Random Forest Regressor	Achieved competitive predictive performance using 4703 complexes; dataset split into training, validation, and testing.	Limited to the structures available in the Protein Data Bank (PDB).
Kathuria et al. (2022) [291]	Classification of biodegradable starch films	Biodegradable starch films	k-Nearest Neighbor (KNN)	Identified optimal film formulation with WVP 1.21 × 10^−10^, TS 2.34 MPa, thickness 0.193 mm.	Limited dataset of 12 films.
Bejagam et al. (2022) [294]	Biocomposites for automotive applications	Wheat straw-filled polypropylene	Polynomial Regression, Artificial Neural Networks (ANNs), SVM	SVM provided the best predictive model for mechanical properties; significant variation in composite properties noted.	Reliance on specific material formulations may limit broader applicability.
Xing et al. (2002) [298]	Predicting molecular weight of polycaprolactone	Polycaprolactone (PCL)	ANN, SVM	SVM was superior for predicting molecular weight based on synthesis parameters; confirmed effectiveness in polymerization.	Focused only on PCL and its synthesis parameters.
Saber et al. (2023) [304]	Optimizing pullulan biosynthesis	Pullulan (from *Aureobasidium pullulans*)	Decision Tree Learning, Taguchi Method	Achieved a pullulan yield of 7.23% with reduced sucrose; optimal conditions identified.	Specific to one strain of fungus; broader applicability needs exploration.
Berger et al. (2020) [320]	Converting orange peels into biodegradable polymers	Orange peels	Decision Tree	Identified optimal production conditions for bioplastics; effective analysis of production variables.	Limited to orange peel feedstock; may not apply to other materials.
Bejagam et al. (2022) [294]	Predicting melting temperatures of PHAs	Polyhydroxyalkanoates (PHAs)	ML models	Developed ML models predicting melting temperature and facilitating polymer design optimization.	Limited dataset for training models; may affect accuracy.
Bohar et al. [327]	ML and additive manufacturing for mechanical strength prediction in FDM-printed components	PEEK (Polyether ether ketone)	Support Vector Regression (SVR), Random Forest Regression (RFR), Genetic Algorithm (GA)	Accurate tensile strength prediction (deviation < 5%), optimized parameters (66.17 MPa tensile strength).	Limited to FDM-printed PEEK components.
Ergun et al. [328]	Xanthan gum-based foam for insulation and packaging	Xanthan gum, cellulose fiber	Generalized Regression Neural Network (GRNN), multiple ML models	Xanthan gum impacted foam properties, R^2^ > 0.97 for GRNN model, optimized foam properties.	Limited to foam properties and materials studied.
Ergun et al. [329]	Guar gum-based foam for insulation applications	Guar gum, cellulose, boric acid	Multiple Linear Regression (MLR), Gaussian Process Regression (GP)	High prediction accuracy (R^2^ up to 0.99), low density, low thermal conductivity, good mechanical strength.	Focused on limited biopolymer-based foam formulations.
Lofgren et al. [330]	Optimization of AquaSolv biorefinery for lignin	Lignin	Bayesian Optimization, Pareto Front Analysis	Maximized lignin yield and β-O-4 linkages, optimized biorefinery conditions.	Limited to lignin depolymerization and chemical processing.
Ifran et al. [331]	ML model for nutrient release prediction from CRFs	Biopolymer-coated controlled-release fertilizers	Gaussian Process Regression (GPR)	R^2^ = 1, RMSE = 0.003, accurate nutrient release time prediction for CRFs.	Focus on CRFs, not applicable to all fertilizer types.
Champa et al. [332]	Enhancing mechanical properties of PBSA with SWCNTs	PBSA, single-walled carbon nanotubes (SWCNTs)	Polynomial Regression (PR), Support Vector Machines (SVMs), Gradient Boosting (GB), Artificial Neural Network (ANN)	Significant improvement in stiffness, R^2^ values ranging from 0.69 to 0.99 for various mechanical properties.	Variability in model performance based on predicted property.

**Table 2:** Summary of unsupervised learning applications in biopolymer research.

Reference	Focus	Material	Applied Model	Results	Limitations
Grishanovich et al. (2024) [333]	Classifying the degree of lignin alteration using solid-state NMR spectroscopy.	Technical lignins	Hierarchical Cluster Analysis (HCA) on solid-state NMR spectra	Method effectively classifies lignin alterations, addressing gaps in correlating dissolved and solid lignins.	Limited to the accuracy of solid-state NMR and its analysis.
Ireddy et al. (2024) [334]	Analyzing PHA film surfaces using AFM and ML algorithms for classification.	Polyhydroxyalkanoate (PHA) films	Unsupervised ML algorithms; benchmarking 12 clustering algorithms; 1D Fourier Transform (FT)	The 1D FT yielded the highest accuracy for film classification. Insights provided on algorithm performance and data impact, along with a preliminary ML tool for surface investigation.	Focused on specific attributes; performance may vary with different film compositions.
Mulrennan et al. (2022) [336]	Predicting mechanical strength of PLA using real-time sensor data.	Polylactide (PLA)	Multivariate regression methods, including partial least squares (PLS), Random Forest (RF), SVR	Combining NIR and conventional sensor data enhanced predictions; RF and SVR showed superior reliability. Nonlinear methods outperformed linear methods.	Method complexity and need for real-time monitoring may limit applicability in practice.
Zhang et al. (2021) [339]	Identifying DNA-binding proteins using optimized features and ensemble learning.	DNA-binding proteins	ML algorithms, LASSO for feature selection, ensemble learning methods	Achieved high accuracy (86.98% and 88.9%) in five-fold cross-validation; effective prediction methodology.	Requires extensive dataset for robust validation; may not generalize to all protein types.
Qiao et al. (2001) [341]	Mapping protein molecular surfaces using SOM for visualization.	Protein molecular surfaces	Kohonen self-organizing map (SOM)	Provides a novel method for the visual comparison of molecular features, effectively addressing complex interrelationships in proteins.	SOM’s effectiveness may vary based on input feature quality.
Jiang et al. (2013) [342]	Improving cis-regulatory element modeling using the CSM model with PWMs.	Transcription factors	Consensus scaffolded mixture (CSM) position weight matrix model with EM algorithm	CSM model showed superior performance compared to other mixture models in 83% of cases, enhancing binding site prediction for transcription factors.	Limited to specific datasets; generalizability to other transcription factors may vary.
Hasan et al. (2014) [344]	Review of motif discovery methods in biological sequences.	Biological sequences	Various data mining techniques, including GYM, modified prefix span method, and grammar-based motif extraction	Identified methodologies improved motif detection rates while addressing training set and support threshold challenges.	Limited exploration of all possible algorithms; focus on recent developments.
Yousef et al. (2016) [346]	Enhancing kNN classification with a new distance metric learning approach.	Plant microRNA species	Ensemble clustering kNN classifier (EC-kNN)	EC-kNN consistently outperformed traditional classifiers, reducing data complexity and improving accuracy through co-clustering distance definitions.	Relies on labeled examples, limiting application to well-characterized datasets.
Wei et al. (2022) [348]	Addressing data limitations in biopolymerization modeling using ML.	Biopolymers	Variational autoencoders, generative adversarial networks (GANs), Random Forest (RF), ANN	Data augmentation improved regression model performance significantly, with RF achieving an R^2^ of 0.94 on the training set and 0.74 on the test set.	Dependence on quality of augmented data; may not fully replicate real-world variability.
Eswaran et al. (2022) [349]	Developing a structural dataset for lignin to facilitate computational research.	Milled wood lignin	Dataset creation and analysis	LGS dataset includes 60,000 structures with 90% accuracy in reflecting experimental properties, serving as a crucial resource for lignin chemistry research.	Limited by existing experimental data and the accuracy of generated structures.
Abreu et al. (2009) [350]	Investigating biohydrogen production from arabinose using anaerobic sludges.	Anaerobic sludges	Modified Gompertz equation for estimating hydrogen production parameters	Higher pH levels correlated with increased hydrogen production; G2 sludge showed the highest yields and efficiency. Strong correlations observed in fermentation pathways.	Specific to arabinose and pH conditions; results may not generalize to other substrates.
Fredricks et al. (2023) [351]	Analyzing biopolymers as sustainable alternatives to fossil-based plastics.	Cellulose, chitin, protein beta-sheet structures	Structural analysis and processing methods for biopolymers	Discusses mechanical properties, processing techniques, and the potential of biopolymers in promoting a circular economy.	Emphasis on selected biopolymer classes; further research needed for broader applicability.

**Table 3:** Summary of deep learning applications in the design of biopolymers.

Reference	Focus	Material	Dataset	Applied Model	Results	Limitations
Khare et al. [387]	Predicting thermal stability	Collagen triple helices	Amino acid sequences with experimental thermal stability data	Transformer models	Small transformer model achieved similar accuracy to larger ProtBERT while using fewer parameters; good performance against experimental data.	Limited to small datasets.
Bandyopadhyay et al. [388]	Exploring conformational landscapes	Mini-proteins and peptides	Molecular dynamics simulation data	Autoencoders	Method outperforms traditional techniques, providing optimized views of protein dynamics and folding pathways.	None specified.
Sadeghi et al. [389]	Designing DNA-stabilized silver nanoclusters	Silver nanoclusters (AgN-DNAs)	DNA sequences with fluorescence properties	Variational autoencoders	Enables multiobjective design for enhanced fluorescence properties and automatic feature extraction; improves on traditional manual engineering methods.	None specified.
Satteri et al. [390]	Inverse design of polymers	Polymers	Materials data with polymer properties	Data-driven approaches	Highlights strategies like high-throughput virtual screening and generative models; discusses optimization challenges.	Challenges in data-driven optimization discussed.
Baldizon et al. [391]	Classifying DNA molecules	Linear and circular DNA	Noisy data from solid-state nanopore experiments	LSTM models, PCA	LSTM achieved highest accuracy (80%) for noisy data classification from solid-state nanopore experiments.	Limited to noisy data context.
Noor et al. [392]	Predicting molecular weight of biopolymers	ε-caprolactone biopolymers	Reaction temperature, time, and molecular weight data	NNs	Accurate predictions of biopolymer molecular weight; demonstrated potential for controlling quality in biopolymerization processes.	Focused on a specific biopolymer process.
Leal et al. [393]	Force sensor development	Hydroxypropyl cellulose (HPC)	RGB color responses of HPC sensors under varying force and concentration	CNN	Achieved a mean squared error of 0.037; highest sensitivity noted at specific HPC concentrations.	None specified.
Salma et al. [394]	Drug release and skin permeation	Piroxicam films from chitosan and xanthan gum	Drug release and permeation data for various formulations	Deep learning, ML	DNN achieved high accuracy; optimal formulation reached 99.97% drug release.	None specified.
Araujo et al. [395]	Thermal degradation of chitosan	Chitosan	Thermogravimetric analysis data	Multilayer perceptron (MLP)	MLP effectively quantified contributions from various kinetic models; lowest residual error recorded.	None specified.
Wong et al. [396]	Biopolymerization performance	ε-caprolactone	Biopolymerization data with molecular weight measures	Multilayer feedforward NN	Identified effective training algorithms; MAPE values for various molecular weights.	None specified.
Laycock et al. [397]	Computational methods in biopolymer design	Biodegradable and bioderived polymers	Computational modeling data for polymeric materials	Multiscale simulations, AI	Integrated framework enhances design flexibility and predicts effects of modifications before testing.	None specified.
Kartal et al. [398]	Thermal degradation of biomass biopolymers	Hemicellulose, cellulose, lignin	Proximate analysis data and thermal degradation behavior	ANN	Excellent performance with R^2^ values over 0.998; allows for immediate calculations of biopolymer fractions in degraded biomass.	Complexity in biomass characterization remains.
Bin et al. [152]	ML in algae-derived biopolymers	Algae-based biopolymers	Material properties and 3D printing process parameters	ML	Highlights potential for sustainable manufacturing and improved mechanical properties; discusses applications in 3D printing.	Technical challenges in material properties optimization.
Asgharzadeh et al. [402]	Deep learning for confocal microscopy	Biopolymer networks	3D confocal microscopy images of biopolymer networks (transformed to 2D slices)	Encoder-decoder network	Achieved a dice score of 0.88 in segmentation tasks; extensive training dataset created from 3D to 2D transformations.	None specified.
Leng et al. [403]	Modeling biopolymer gel behavior	Fibrin, collagen	Strain energy data from fiber networks under biaxial deformation	FCNN	Successfully predicts strain energy derivatives; integrated into finite element software for nonlinear elasticity problems.	None specified.
Nobrega et al. [404]	Biodegradable starch-based films	Starch-based films	Mechanical and barrier property data with additive effects	Self-organizing maps (SOMs), MLP	Achieved high correlation (max error 24%) in predicting mechanical and barrier properties; emphasizes role of glycerol.	Further data needed to improve model accuracy and compatibility.
